# Effect of live yeast *Saccharomyces cerevisiae* supplementation on the performance and cecum microbial profile of suckling piglets

**DOI:** 10.1371/journal.pone.0219557

**Published:** 2019-07-22

**Authors:** Tadele G. Kiros, Diana Luise, Hooman Derakhshani, Renee Petri, Paolo Trevisi, Romain D’Inca, Eric Auclair, Andrew G. van Kessel

**Affiliations:** 1 University of Saskatchewan, Department of Animal and Poultry Science, Saskatoon, Saskatchewan, Canada; 2 Department of Agricultural and Food Sciences, University of Bologna, Bologna, Italy; 3 Department of Animal Science, University of Manitoba, Winnipeg, Manitoba, Canada; 4 Phileo-Lesaffre Animal Care, Marcq-en-Baroeul, France; University of Illinois, UNITED STATES

## Abstract

One mechanism through which *S*. *cerevisiae* may improve the performance of pigs is by altering the composition of the gut microbiota, a response that may be enhanced by early postnatal supplementation of probiotics. To test this hypothesis, newborn piglets (16 piglets/group) were treated with either *S*. *cerevisiae* yeast (5 x 10^9^ cfu/pig: Low) or (2.5 x 10^10^ cfu/piglet: High) or equivalent volume of sterile water (Control) by oral gavage every other day starting from day 1 of age until weaning (28±1 days of age). Piglet body weight was recorded on days 1, 3, 7, 10, 17, 24 and 28 and average daily gain (ADG) calculated for the total period. At weaning, piglets were euthanized to collect cecum content for microbial profiling by sequencing of the 16S rRNA gene. ADG was higher in both Low and High yeast groups than in Control group (*P*<0.05). Alpha diversity analyses indicated a more diverse microbiota in the Control group compared with Low yeast group; the High yeast being intermediate (*P* < 0.01). Similarly, Beta diversity analyses indicated differences among treatments (*P* = 0.03), mainly between Low yeast and Control groups (*P* = 0.02). The sparse Partial Least Squares Discriminant Analysis (sPLS-DA) indicated that Control group was discriminated by a higher abundance of *Veillonella*, *Dorea*, *Oscillospira* and *Clostridium*; Low yeast treated pigs by higher *Blautia*, *Collinsella* and *Eubacterium*; and High yeast treated pigs by higher *Eubacterium*, *Anaerostipes*, *Parabacteroides*, *Mogibacterium* and *Phascolarctobacterium*. Partial Least Squares (PLS) analysis showed that piglet ADG was positively correlated with genus *Prevotella* in High yeast group. Yeast supplementation significantly affected microbial diversity in cecal contents of suckling piglets associated with an improvement of short chain fatty acid producing bacteria in a dose-dependent manner. In conclusion, yeast treatment improved piglet performance and shaped the piglet cecum microbiota composition in a dose dependent way.

## Introduction

The use of *Saccharomyces cerevisiae* (*S*. *cerevisiae)* yeast as a feed additive for farm animals has increased enormously in the last decade, especially after the ban of antibiotic growth promoters in Europe. In ruminants, *S*. *cerevisiae* is reported to increase feed efficiency [1] and improve milk production, as well as milk quality [2,3]. Data from monogastric animals have been contradictory; some reporting no effect on feed intake or body weight gain of sows [4] or weanling pigs [5], while others have reported improved milk quality in sows [6] and increased daily feed intake and body weight gain in weanling pigs [7,8,9] as well as the increasing resistance against Enterotoxigenic *Escherichia coli* (ETEC) F4 or *Salmonella* infection in piglets [10,11,12]. Similarly, daily supplementation of broiler chicken with live *S*. *cerevisiae* increased feed intake, body weight gain, feed conversion ratio, and improved carcass quality [13,14]. Despite the contradictory results coming from monogastric animal studies, the use of live yeast as a feed additive in the livestock industry is on the rise due to the general understanding that probiotics, including live yeast supplementation, improve the health and performance of farm animals [4,5]. However, the mode of action by which live yeast supplementation improves the overall health and performance of farm animals is not yet fully elucidated. Since the pig gut microbiota is considered to be unstable during the first 3 to 4 weeks of age [15], early supplementation of suckling piglets with a yeast probiotic may improve the stability of the gut microbiota before weaning and improve gut health and performance in newborn piglets. The objective of this study is, therefore, to evaluate the effect of *S*. *cerevisiae* (Actisaf CNCM I-4407) supplementation on the performance and composition of the cecum microbiota of piglets before weaning.

## Material and methods

### Ethics statement

The study was conducted, and animals handled according to the regulations and guidelines provided by the Canadian Council on Animal Care for humane animal use and approved by the University of Saskatchewan Animal Research Ethics Board under animal use protocol number AUP-20110132.

### Experimental design

**Fig 1:**
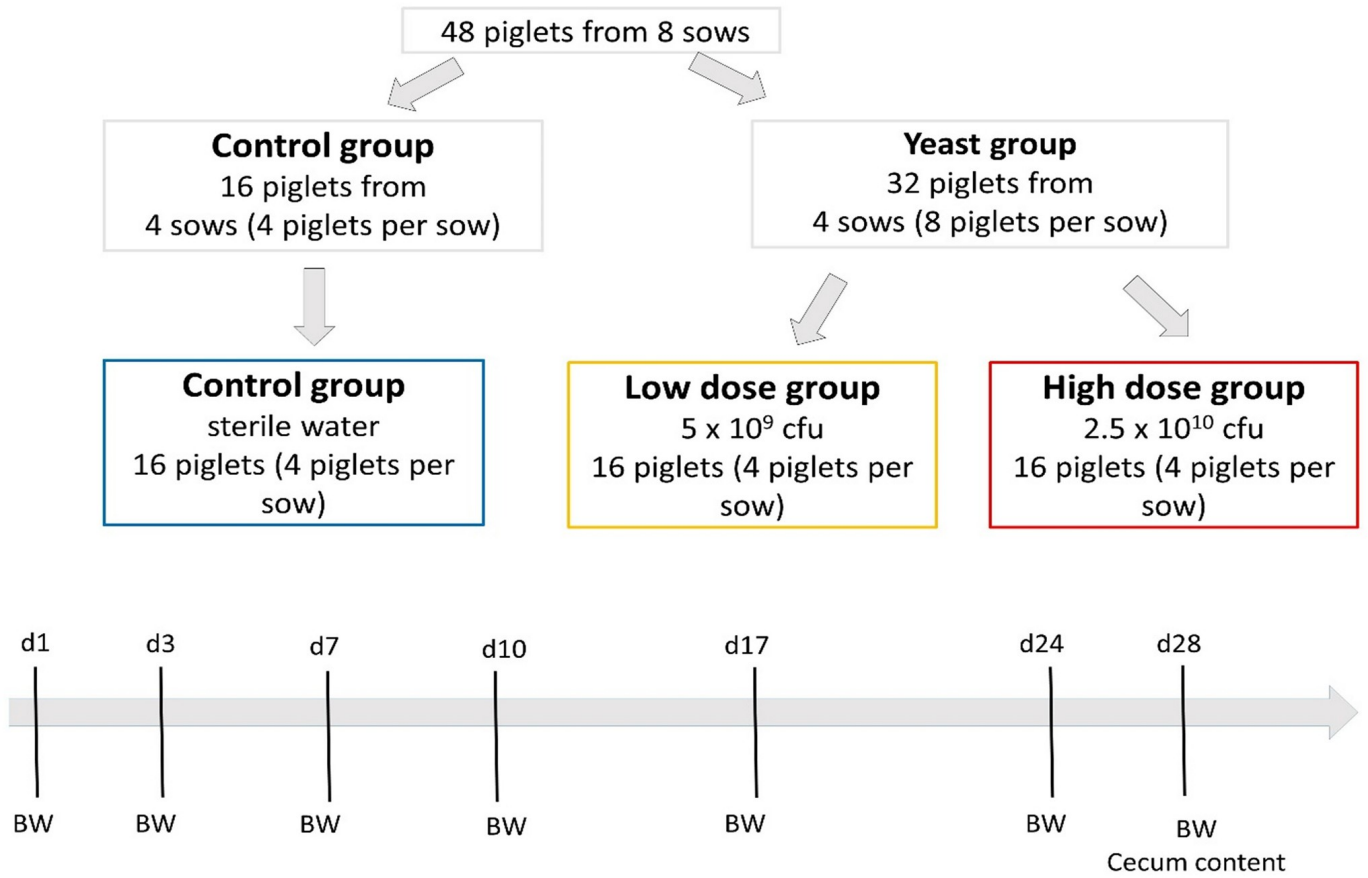
Experimental design. Eight sows were randomly selected among the newly farrowed sows at the Prairie Swine Center (University of Saskatchewan, Canada) and assigned to either Control or Yeast group, 4 sows per group balanced for parity. Eight healthy looking piglets (balanced for sex and body weight) were chosen from each sow in the Yeast group (n = 32) and assigned either to Low dose yeast group (n = 16; 4 piglets per sow) or to High dose yeast group (n = 16; 4 piglets per sow). Four healthy looking piglets (balanced for sex and body weight) were chosen from each sow in the Control group and ear tagged for individual identification (n = 16; 4 piglets per sow). Low dose piglets received the regular dose (5 x 10^9^ cfu/piglet; Low) of *S*. *cerevisiae* yeast (Actisaf, CNCMI-4407; Phileo-LeSaffre Animal Care, Marcq-en-Baroeul, France) recommended by the manufacturing company, while High dose yeast piglets) received a higher dose of yeast (2.5 x 10^10^ cfu/piglet; High). Yeast solutions were prepared by mixing yeast powder in sterile water and administered to piglets by oral gavage using a 10 mL syringe. Piglets were individually weighted at days 1,3,7,10,17,24,28 of age. On weaning day, piglets were euthanized.to collect the cecum content to analyses the microbial profile.

Fig 1 summarizes the experimental design. Eight sows were randomly selected among the newly farrowed sows at the Prairie Swine Center (University of Saskatchewan, Canada) and assigned to either Control or Yeast group, 4 sows per group balanced for parity. Control and Yeast group sows and their newborn piglets were housed in the same farrowing room on opposite side of the room to avoid cross contamination between Yeast and Control groups. Eight healthy looking piglets were chosen from each sow in the Yeast group and ear tagged for individual identification. Four piglets from each sow in the Yeast group (n = 16) received the regular dose (5 x 10^9^ cfu/piglet in a total volume of 3 mL; Low) of *S*. *cerevisiae* yeast (Actisaf, CNCMI-4407; Phileo-LeSaffre Animal Care, Marcq-en-Baroeul, France) recommended by the manufacturing company, while the remaining 4 piglets (n = 16) received a higher dose of yeast (2.5 x 10^10^ cfu/piglet in a total volume of 6 mL; High). Yeast solutions were prepared by mixing yeast powder in sterile water and administered to piglets by oral gavage using a 10 mL syringe. Four piglets from each of the Control group sows (n = 16) were also ear tagged and received equal volume of sterile water by gavage (Control). To the extent possible, piglets in each treatment group were balanced for weight and sex. Yeast dietary supplementation and the Control treatment were administrated every other day starting from day (d) 1 of age until weaning (d28 ± 1). Control and yeast treated piglets were deliberately selected from different litters to avoid cross-contamination of Control piglets by yeast shedding from yeast supplemented piglets if they were to be kept with the same sow in the same pen throughout the study period. During the lactation period, sows had free access to feed and water. Diet was formulated to meet or exceed the National Research Council [16] nutrient requirements for lactating sows. However, lactating sows did not get any yeast supplementation. Piglets had free access to water and no creep-feed was given during pre-weaning. The farrowing room temperature was maintained at 23 °C and heat lamps were used the first week after birth. After one week of age, the heat lamps were used only at night.

### Sample collection

Piglet body weight (BW) was recorded on days 1, 3, 7, of age and then every week until weaning (day 28 ± 1of age). Average daily gain (ADG) was individually calculated by dividing the body weight difference between the final weight and the initial weight to the number of days each pig was in the study (Fig 1). All information on piglets’ weight at the different time points and piglets’ ADG is included in S1 Table. At weaning, piglets were humanely killed by captive bolt stunning and pitching. From each pig (16 piglets/group) caecal content was collected and immediately stored at -20°C until analysis.

### Bacterial DNA extraction and purification

Total bacterial DNA was extracted from 350 mg cecal contents by bead-beating method using the 25:24:1 phenol/chloroform/isoamyl alcohol extraction technique as previously described [17]. Extracted DNA was re-suspended in 500 μL nucleic acid free water and purified using the bead based ChargeSwitch gDNA purification kit (Invitrogen) according to the manufacturer’s protocol. The concentration of the DNA eluted in 200 μL of elution buffer was measured and purity determined by absorbance at 260 and 280 nm using the NanoDrop; ND-1000 Spectrophotometer. DNA samples were subjected to a deep sequence analysis (10,000 reads per sample) at the Molecular Research (MR DNA) laboratory in Shallow water, TX, USA.

### 454 pyrosequencing

Amplicon pyrosequencing (bTEFAP) originally described [18] was utilized for deep sequence analysis of intestinal content samples. Briefly, the 16S universal Eubacterial primers 27F (AGRGTTTGATCMTGGCTCAG) and 519R (GTNTTACNGCGGCKGCTG) which amplify a sequence spanning the V1-V3 hypervariable regions were used together with short chain unique DNA sequence tags (barcodes) [19]. Around 100 ng (1μL) pure DNA was used per 50μL of PCR reaction. A single-step 30 cycles PCR using HotStarTaq Plus Master Mix Kit (Qiagen, Valencia, CA) was used under the following conditions: 94°C for 3 minutes, followed by 28 cycles of 94°C for 30 seconds; 53°C for 40 seconds and 72°C for 1 minute. The final elongation step was for 5 minutes at 72°C. Following PCR, all amplicon products from different samples were pooled in equal concentrations and purified using Agencourt Ampure beads (Agencourt Bioscience Corporation, MA, USA). Samples were sequenced utilizing Roche 454 FLX titanium instrument and reagents (Roche, Nutley, New Jersey) following the manufacturer’s instructions.

### Sequence processing

Sequence data derived from the sequencing process were subjected to a stringent quality control screening processes. Briefly, sequences were depleted of barcodes and primers, and then short sequences < 150bp, sequences with ambiguous base calls, and sequences with homopolymer run exceeding 6 bp were removed. The remaining reads were further screened and those with more than 3 consecutive bases with quality scores below 25 were truncated. Sequences were assigned to Operational Taxonomic Units (OTUs) using subsampled open-reference; average read length of OTUs was 436 nucleotides. OTU- picking was carried out using UCLUST with 97% sequence similarity on QIIME (v1.9.1). Singleton OTUs were removed and taxonomies were assigned to the representative sequence of each OTU using UCLUST and aligned with the latest Greengenes Core reference database (version 13_8) [20] using PyNAST algorithms [21]. Data were chimera checked using the Blast fragments approach [22] in QIIME Phylogenetic tree was built with FastTree 2.1.3 [23] for further comparison between microbial communities.

### Bioinformatics and statistical analysis

Body weight was analyzed using an ANOVA mixed model in which age and treatment were included as fixed effects, while sow was included as random effect, and piglets as repeated measurement (by including random intercept terms) to account for multiple observations within the same sow or within the same piglets. ADG was analyzed using an ANOVA mixed model in treatment was included as fixed effects, while sow was included as random effect, and piglets as repeated measurement. Tukey’s honest significance test was carried out at a 95% confidence level to test multiple comparison among group. Effects were considered significant when *P* < 0.05. Statistical analysis was performed with R statistical software (v.3.3.0) [24] using the ª*lme4*” and “emmex” packages.

The singletons and OTUs with relative abundance across all samples below 0.005% were removed as recommended by Bokulich [25]. Four samples that reported low sequencing yield (less than 4000 reads after quality check) were excluded from further analysis (three samples from CON group and one sample from Low yeast group). Number of sequences per sample ranged from 3982 to 18,200 and sequences were classified into 744 representatives OTUs. Biostatistics on OTUs were performed using phyloSeq [26], Vegan [27] and mixOmix [28] packages in R software (v.3.3.0) [24].

The richness (observed OTUs) and alpha diversity indices (Shannon and Chao indices (Chao, 1984 [29]) were calculated on raw data and comparison between treatments were tested using a mixed model including treatment as fixed factor and sow as random factor. Bray-Curtis Dissimilarity was calculated and used to compare the treatment differences in beta diversity using a permutational MANOVA (Adonis and pairwise.adonis procedure) including treatment and sow as factors. Non-Metric Multidimensional Scaling Plots (NMDS) based off Bray-Curtis Dissimilarity distances were visualized in R software [24] using ggplot2. Beta diversity ordination (Bray Curtis distance matrix) was calculated after rarefaction correction.

Taxonomic differences among groups were tested using Wilcoxon signed-rank test on OTUs aggregated data at Genus level. *P*-values were corrected for FDR correction. *P*-value < 0.05 was considered as significant and *P*-value < 0.1 was considered a trend of significance. Furthermore, to further investigate the differences in cecum microbiota composition between groups, the sparse Partial Least Squares Discriminant Analysis (sPLS-DA) was performed [30, 31]. It performs a variable (OTUs aggregated at Genus level) selection with Lasso penalization method. The optimal number of components were selected based on the averaged balanced classification error rate with centroids distance over 50 repeats of a 5-fold cross-validation of a sPLS-DA model with 3 components (using “perf” function). The optimal number of selected variables for each component was then chosen based on the lowest average balanced classification error rate with centroids after tuning of the sPLS-DA model (function tune.spldsda) using the selected number of components and 5-fold cross-validation with 50 repeats. Stability frequency scores of the selected OTUs were calculated (function perf) on the final sPLS-DA model with 5-fold cross-validation and 50 repetitions [30]. Individual samples were presented on a score plot and were distinguished by treatments with colour and 95% confidence elipses. Discriminant genera were plotted according to their contribution weight to the component 1 of 2 of sPLS-DA and were distinguished by treatments with colours [31].

Finally, we tested the association between cecum microbiota and ADG of piglets of Low and High dose yeast supplemented groups using a Partial Least Squares Analysis (PLS) approaches and the network function [32]. The ‘network’ function calculated a similarity measure between X (OTUs) and Y (ADG values) variables in a pairwise manner. OTUs showing a pairwise associations with scores greater than 0.50 were considered significant.

## Results

### Growth performance

**Fig 2:**
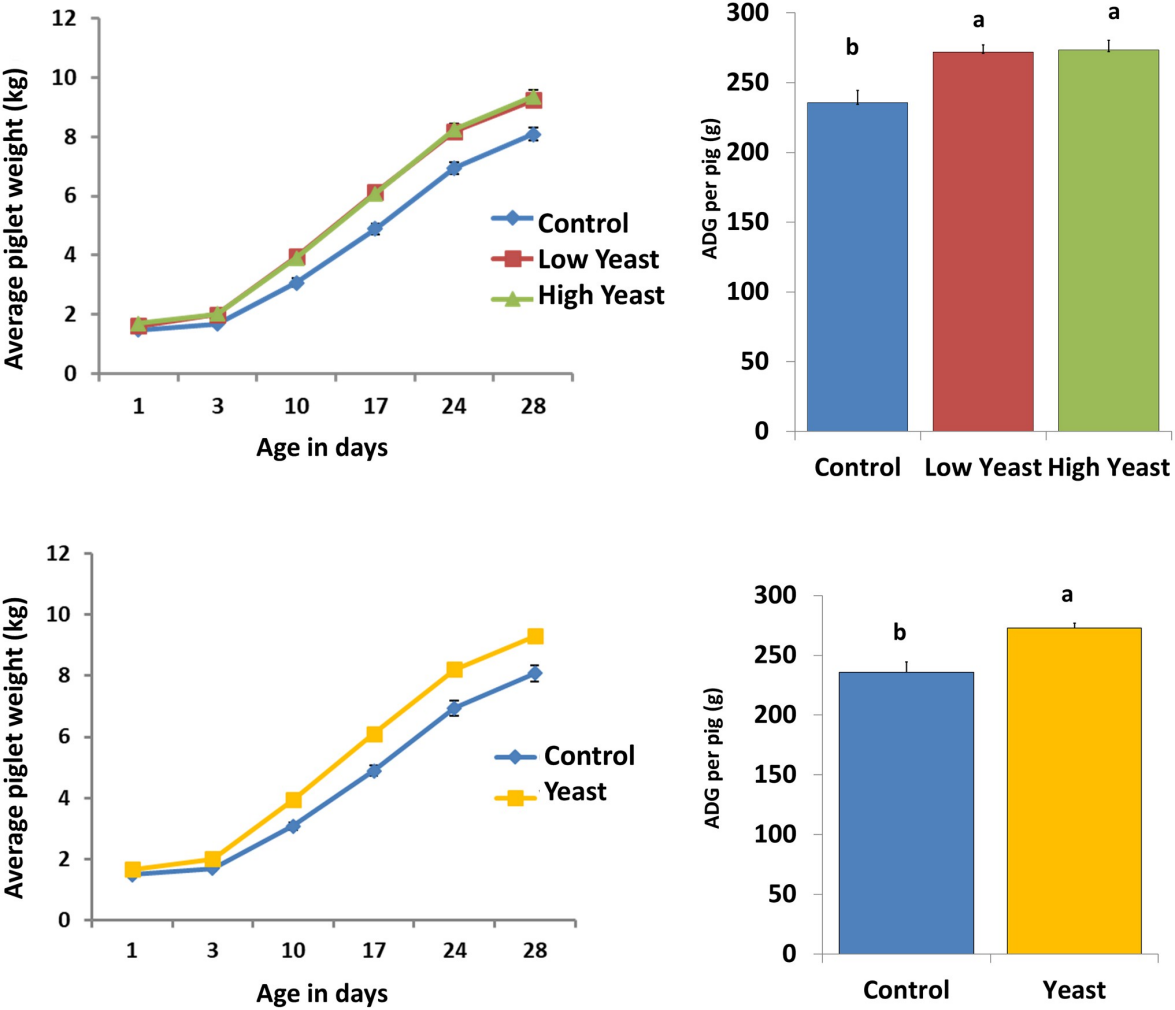
Performance data of piglets treated with two different doses of yeast or water. Piglets were treated by oral gavage with either 5 x 10^9^ cfu (Low) dose or 2.5 x 10^10^ cfu (High) dose of live yeast or sterile water (Control) every other day starting from day one of age until weaning. Body weight was recorded at regular intervals and presented either as average piglet weight in kilograms (**A**) or average daily weight gain in grams (**B**). Error bars represent SEM. Pooled data for low dose and high dose yeast groups were also compared against control piglets (**C, D**). Error bars represent SEM; bars with different letters are significantly different at *P* < 0.05.

Age and treatment significantly influenced the piglet body weight (*P* < 0.001). There was no significant difference in body weight between the different treatment groups on day 1 of age (*P* > 0.1). Piglets that received Low or High dose yeast supplements were heavier than Control piglets by 10 days of age and remained heavier until the end of the study (*P* < 0.05) (Fig 2A). The overall ADG was higher in both the High and Low dose yeast-treated piglets as compared to the Control piglets (*P* = 0.017 and *P* = 0.016, respectively) (Fig 2B). As there was no difference in performance between the High dose and Low dose of yeast treated piglets, we pooled the data for the low dose and high dose yeast groups and compared with the control group (Fig 2C and 2D). Yeast treatment significantly improved (*P < 0*.*05*) body weight and ADG.

### Microbial diversity and species richness

**Fig 3:**
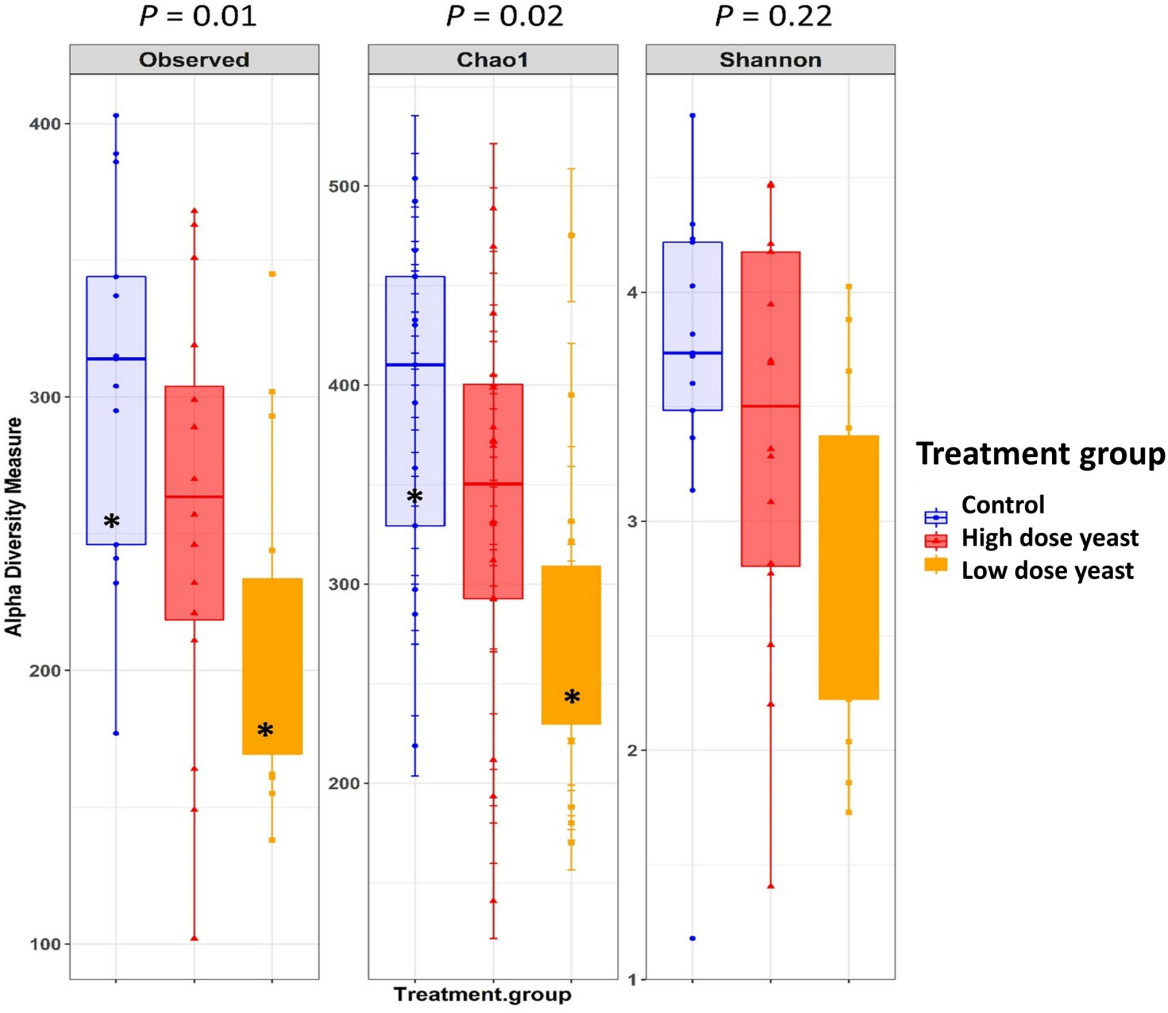
Alpha diversity indices in cecum content of piglets treated with two different doses of yeast or water. Piglets were treated by oral gavage with either 5 x 10^9^ cfu (Low) dose or 2.5 x 10^10^ cfu (High) dose of live yeast or sterile water (Control) every other day starting from day one of age until weaning. Piglets were euthanized on weaning day and cecum content collected for microbiota analysis. Species richness and diversity index were measured with three different matrices: Observed OTUs, Chao1 and Shannon index. The number of piglets per group were respectively: 13 in Control; 15 in Low yeast; 16 in High yeast. * stands for *P* < 0.05.

Table 1 details the various microbial diversity indices and statistical differences among treatments. Number of observed OTUs and Chao richness estimate has indicated that the microbial communities in the hind gut of the Control piglets were more rich in terms of bacterial species present than the community in the Yeast treated piglets (*P* < 0.05), while no difference was observed in Shannon diversity index. As shown in Table 1 and Fig 3, the lower dose yeast dietary supplementation reduced the number of observed OTUs and Chao’s richness estimate compared to Control (*P* = 0.01). No significant difference between the High dose yeast dietary supplementation and Control were reported for number of observed OTUs and alpha diversity indices.

**Table 1:** Microbial diversity and species richness in cecum content of suckling piglets treated with two different doses of yeast or water.

	Treatments^1^					*P*-value			
Item^2^	Control	Low Yeast	High Yeast	Yeast (both)	SE	Total	Control vs Low Yeast	Control vs High Yeast	Control vs Yeast
Nseq	301.77	218.17	257.03	237.60	14.70	0.01	0.01	0.16	0.01
Chao 1	382.57	283.64	337.14	310.39	23.32	0.02	0.01	0.30	0.02
Shannon	3.48	3.04	3.34	3.19	0.17	0.22	0.17	0.79	0.20

^1^Treatments: Control = no yeast; Low yeast = 5 x 10^9^ cfu; High yeast = 2.5 x 10^10^ cfu of live *S*. *cerevisiae*; Yeast (both) = data from Low dose yeast and High dose yeast treated pigs pooled together. The number of piglets per group were respectively: 13 in Control; 15 in Low yeast; 16 in High yeast.

^2^Diversity indices: nseqs = number of unique sequences; Choa 1 = chaos’s estimate of richness; Shannon = Shannon index.

### Microbial community structure

**Fig 4:**
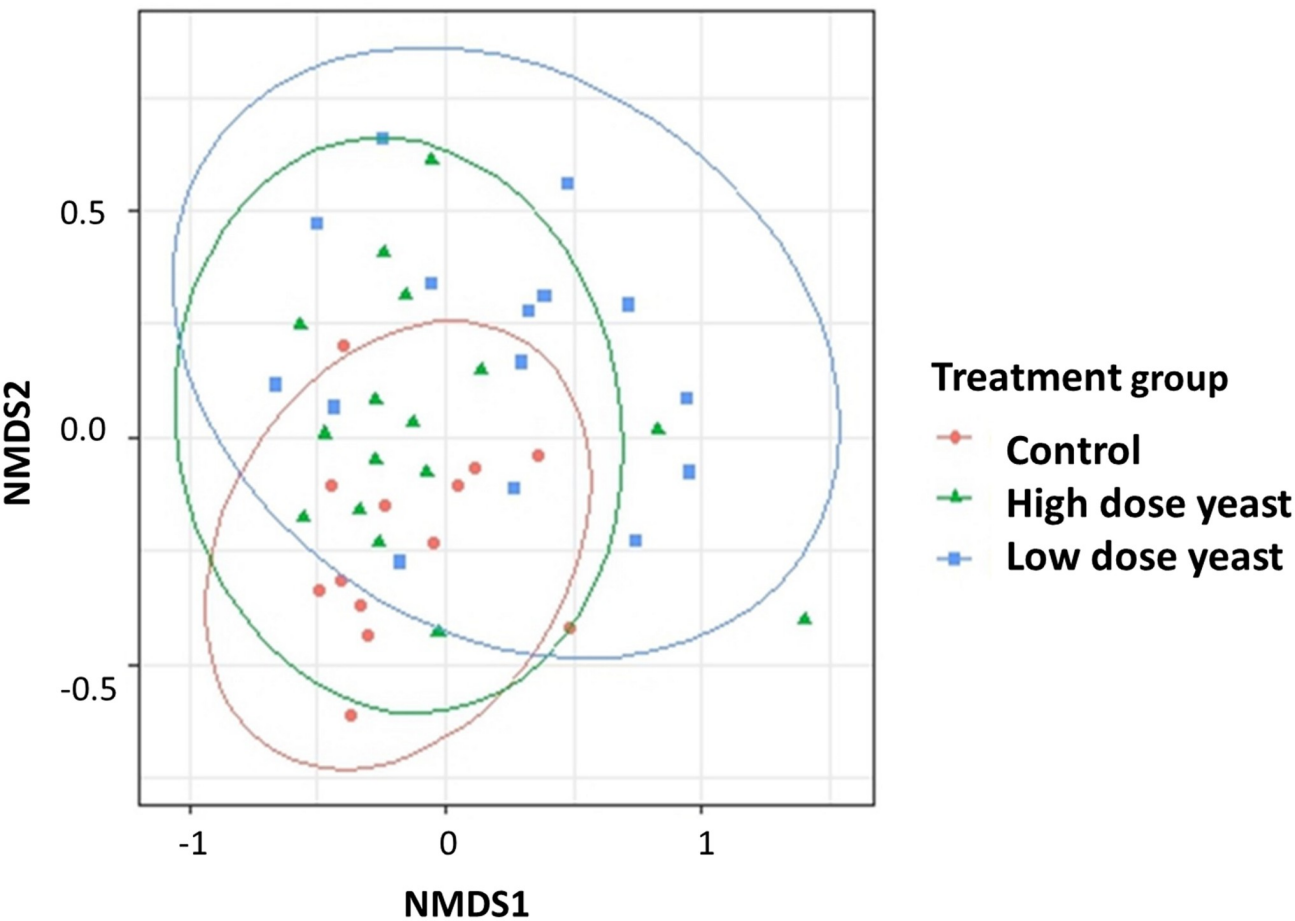
Non-Metric Multidimensional Scaling Plots (NMDS) based on Bray-Curtis Dissimilarity distances in cecum content of piglets treated with two different doses of yeast or water. Piglets were treated by oral gavage with either 5 x 10^9^ cfu (Low) dose or 2.5 x 10^10^ cfu (High) dose of live yeast or sterile water (Control) every other day starting from day one of age until weaning. Piglets were euthanized on weaning day and Cecum content collected for microbiota analysis. In the NMDS plot, each point represents the gut microbiota of a pig and colors and shapes visualize each Treatment. The number of piglets per group were respectively: 13 in Control; 15 in Low yeast; 16 in High yeast.

Multivariate analyses showed treatment was significant in driving diversity assessed on Bray-Curtis Dissimilarity (*P* = 0.03). A significant difference was observed between Control and Low dose yeast supplemented group (*P* = 0.02) and between Low dose and High dose yeast supplemented groups (*P* = 0.05), while no difference was observed between Control and High dose yeast supplemented groups. NMDS plot based on the Bray-Curtis Dissimilarity did not show any distinct clustering pattern between the treatment groups. However, the Low dose yeast supplemented group showed a higher dispersion compared to the Control group (Fig 4). This difference was further supported by the permutational MANOVA test (*P* = 0.02).

### Phylogenetic assignment and discriminant bacterial population

**Fig 5:**
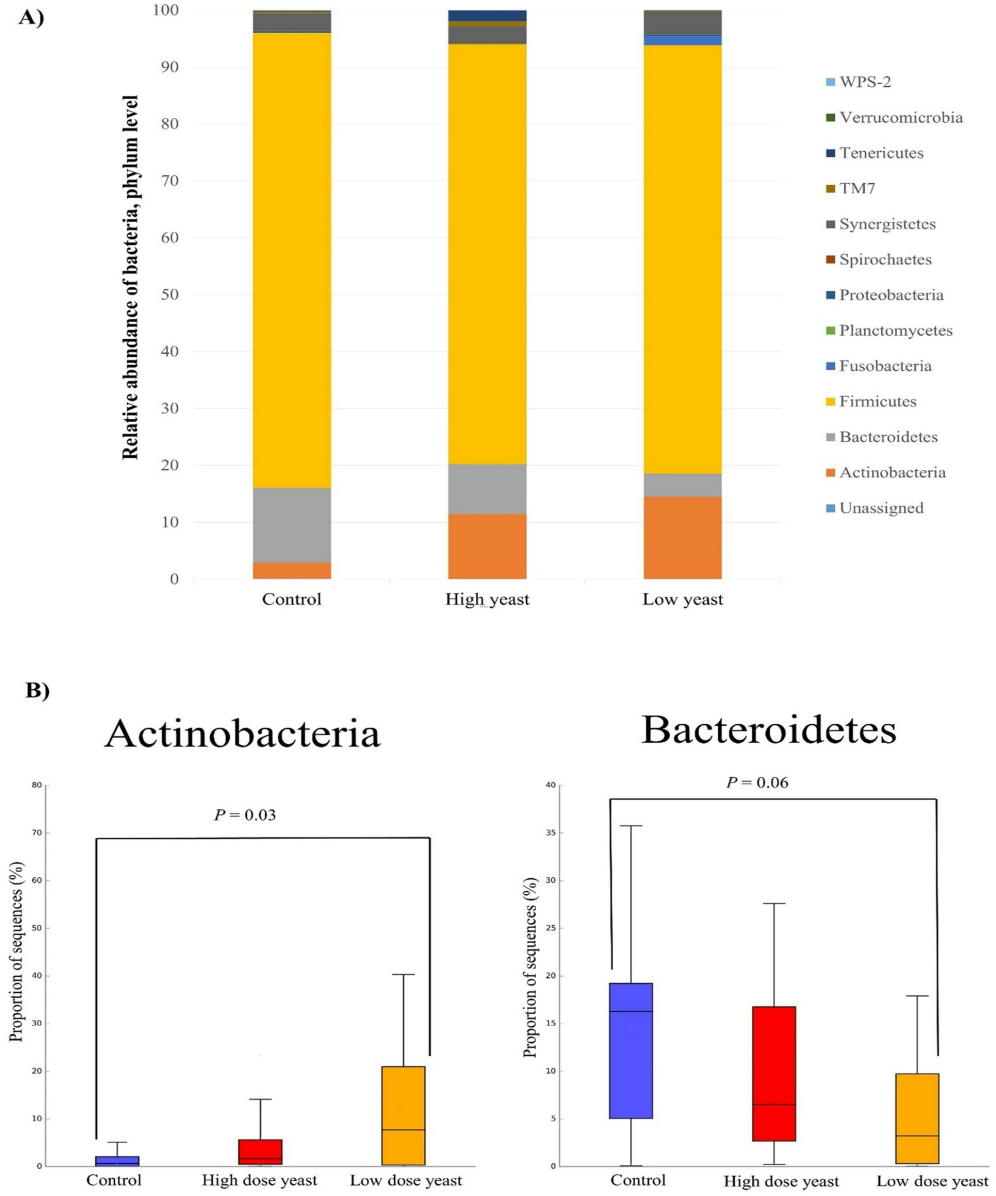
Microbiota differences at phylum level in cecum content of piglets treated with two different doses of yeast or water. Piglets were treated by oral gavage with either 5 x 10^9^ cfu (Low) dose or 2.5 x 10^10^ cfu (High) dose of live yeast or sterile water (Control) every other day starting from day one of age until weaning. Piglets were humanely euthanized on weaning day and Cecum content collected for microbiota analysis. **A**) Bar graphs show the mean relative abundance of each bacterial phyla calculated for each treatment group. **B**) Box plots show the proportion of the two abundant and significantly different bacterial Phyla among groups in the cecum microbiota of sucking piglets. Boxes displayed the interquartile range with line in the median and whiskers showed the minimal and maximal observed data. The number of piglets per group were respectively: 13 in Control; 15 in Low yeast; 16 in High yeast.

Phylum level classification of the bacterial population revealed that more than 90% of the bacteria in the hind gut of neonatal piglets belong to either one of the three major phyla Firmicutes, Bacteroidetes, and Actinobacteria (Fig 5A). There was no significant difference between the groups in the relative abundance of Firmicutes; 83.14% in Control, 82.04% in Low dose yeast, and 81.99% in High dose yeast group. The relative abundance of phylum Bacteroidetes tended to be lower in the Low dose yeast group (4.88%) than in Control group (14.12%) (*P* adj. = 0.06) (Fig 5B), while no significant difference was reported for High dose yeast supplementation (8.39%) compared to Control (9.60%). In contrast a higher proportion of phylum Actinobacteria was reported in the Low dose yeast supplemented group than in Control group (12.01% in Low dose yeast, 2.73% in Control; *P* adj. = 0.03), while no significant difference was reported between High dose yeast supplementation group (8.39%) and Control group (Fig 5B). At Family level, *Ruminococcaceae* (31.6%), followed by *Lactobacillaceae* (16.8%) and *Erysipelotrichaceae* (9.7%) were the most abundant and no significant difference was observed among groups.

At Genus level, *Unclassified Ruminococcaceae* (27.2%), followed by *Lactobacillus* (16.8%) and *Unclassified Clostridiales* (9.2%) were the most abundant. Table 2 reports the significantly different genera between yeast and Control groups and their relative abundance. A trend of significant differences was reported for *Prevotella*, *Unclassified Lachnospiraceae*, *Oscillospira* and *Dorea* genera among groups after the FDR correction (*P* adj. < 0.1). A higher number of different genera abundance can be described without the multiple corrections. Control had higher *Oscillospira* abundance than both Low and High yeast supplemented groups (*P* < 0.05), higher abundance of *Dorea*, *Prevotella*, *Unclassified Lachnospiraceae*, *Acidaminococcus* and *Unclassified Peptostreptococcaceae* than Low yeast supplemented group (*P* < 0.05) and higher abundance of *Veillonella* than High yeast supplemented group (*P* < 0.05). A trend of higher *Dorea* abundance was observed in Control than in High yeast group (*P* = 0.08). Low dose yeast group had higher *Collinsella* than the Control group (*P* = 0.03) and a trend of higher *Blautia* (*P* = 0.07). High dose yeast group had higher *Blautia* (*P* = 0.007) than Control group and a trend of higher *Pseudoramibacter_Eubacterium* and *Roseburia* (*P* < 0.1).

**Table 2:** Microbial diversity at genus level in cecum content of suckling piglets treated with two different doses of yeast or water. Data are reported as Relative abundance (%).

Genus	Treatments^1^				Total		Control vs Low Yeast		Control vs High Yeast	
	Control	Low dose Yeast	High dose Yeast	SE	*P*-values	*P*-values adj.	*P*-values	*P*-values adj.	*P*-values	*P*-values adj.
Prevotella	5.24	0.46	3.07	0.04	0.006	0.083	0.003	0.177	0.235	0.762
Unclassified Lachnospiraceae	1.32	0.60	1.56	0.02	0.014	0.084	0.015	0.155	0.578	0.835
Oscillospira	3.47	1.18	1.50	0.03	0.005	0.085	0.016	0.137	0.029	0.741
Dorea	0.42	0.08	0.12	0.01	0.014	0.090	0.042	0.275	0.075	0.646
Veillonella	0.04	0.01	0.00	0.00	0.013	0.100	0.108	0.561	0.042	0.731
Acidaminococcus	0.02	0.00	0.01	0.00	0.004	0.109	0.013	0.169	0.279	0.605
Unclassified Peptostreptococcaceae	0.11	0.02	0.10	0.01	0.023	0.118	0.012	0.207	0.788	0.976
Clostridium	0.16	0.06	0.01	0.01	0.003	0.167	0.241	0.570	0.053	0.684
Ruminococcus	2.92	1.85	2.06	0.03	0.059	0.218	0.126	0.595	0.092	0.599
Blautia	0.26	0.66	0.80	0.02	0.059	0.235	0.071	0.411	0.007	0.388
Collinsella	2.71	11.97	2.54	0.06	0.131	0.358	0.027	0.199	0.919	1.039
Pseudoramibacter_Eubacterium	0.01	0.04	0.04	0.00	0.232	0.548	0.150	0.601	0.077	0.570
Roseburia	0.00	0.01	0.03	0.00	0.281	0.561	0.457	0.609	0.073	0.762

^1^Treatment: Control = no yeast; Low yeast = 5 x 10^9^ cfu; High yeast = 2.5 x 10^10^ cfu of live *S*. *cerevisiae* per pig every other day. The number of piglets per group were respectively: 13 in Control; 15 in Low yeast; 16 in High yeast

**Fig 6:**
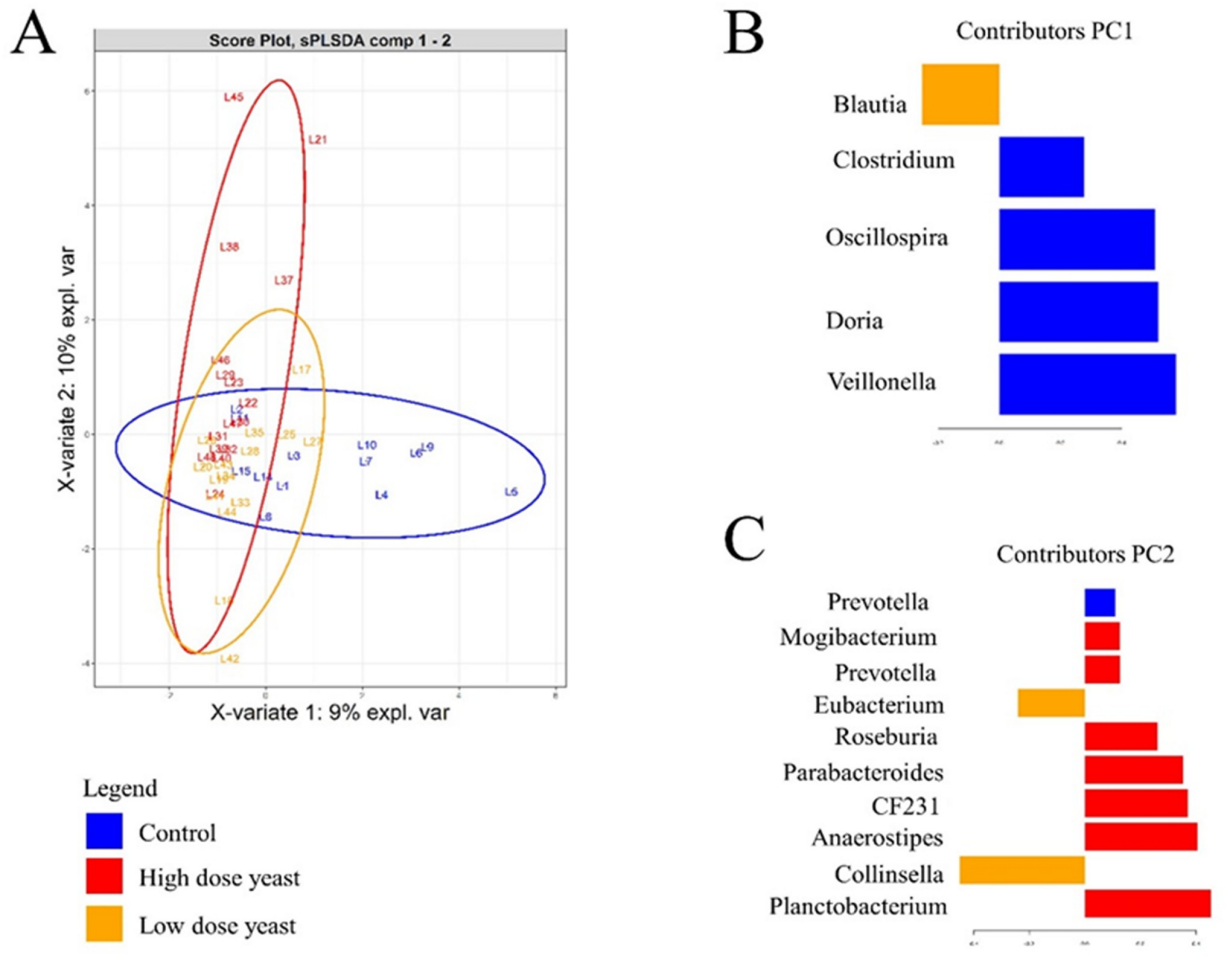
Results of sPLS-DA for microbial diversity at Genus level in cecum content of piglets treated with two different doses of yeast or water. **A)** Individual score plot of the samples along the first two components. **B, C)** Contribution plot represented the contribution of each genus on the first and second component. Genus contribution ranked from bottom (most important) to top. Colors indicate the group in which the feature is most abundant. The number of piglets per group were respectively: 13 in Control; 15 in Low yeast; 16 in High yeast.

The sPLS-DA was applied to identify specific genera that could potentially distinguish among the treatment groups. Fig 6A, 6B and 6C shows the individual score plot and the contribution plots, while Table 3 lists the discriminant genera for the different treatments. As can be seen in Fig 6A the final model resulted in clustering of samples according to treatment groups. The 1st and 2nd latent components contribute towards 9% and 10% of explained variance. The Control group was discriminated by a higher abundance of *Veillonella*, *Dorea*. *Oscillospira*, *Clostridium* and *Prevotella* genera; Low yeast dietary treatment group was discriminated by a higher abundance of *Blautia* and *Eubacterium* genera; High yeast dietary treatment group was discriminated by a higher abundance of *Phascolarctobacterium*, *Anaerostipes*, *CF231*, *Parabacteroides*, *Eubacterium*, *Prevotella and Mogibacterium* genera.

**Table 3:** Discriminat genera in cecum content of sucking piglets treated with two different doses of yeast or water. Results of geneara contribution weight to the components 1 and 2 of sPLS-DA.

Genus	Value.var^1^	Stability^2^	PC^3^	Treatment^4^
Veillonella	0.58	0.76	1	Control
Dorea	0.52	0.80	1	Control
Oscillospira	0.51	0.82	1	Control
Clostridium	0.28	0.45	1	Control
Blautia	-0.25	0.52	1	Low Yeast
Phascolarctobacterium	0.45	0.83	2	High Yeast
Collinsella	-0.45	0.79	2	Low Yeast
Anaerostipes	0.40	0.95	2	High Yeast
Parabacteroides	0.35	0.71	2	High Yeast
[Eubacterium]	0.26	0.74	2	High Yeast
[Eubacterium]	-0.24	0.70	2	Low Yeast
[Prevotella]	0.13	0.53	2	High Yeast
Mogibacterium	0.12	0.63	2	High Yeast
Prevotella	0.11	0.44	2	Control

^1^value.var, expresses the variance explained by the single genera.

^2^Freq, express the frequencies by which the genera were chosen among the 100 repetitions of the cross validation

^3^PC stands for the principal component that discriminate the genera

^4^Treatment: Control = no yeast; Low yeast = 5 x 10^9^ cfu; High yeast = 2.5 x 10^10^ cfu of live *S*. *cerevisiae* per pig every other day

The number of piglets per group were respectively: 13 in Control; 15 in Low yeast; 16 in High yeast

### Microbiota influence on piglet ADG

Influence of microbiota composition on piglet ADG in the Low and High yeast supplemented groups were assessed using the PLS model. No OTUs were positively correlated with the piglets’ ADG in the Low dose yeast group (S2 Table). Thirteen OTUs were positively correlated with the piglets’ ADG (correlation> 0.50) in the High dose yeast group. Three out of thirteen OTUs belong to genus *Prevotella*, one OTU to genus *Blautia*. The remaining nine positively correlated OTUs were assigned only to the Family or Order taxonomy level; four OTUs, belong to Family Ruminococcaceae and three OTUs belong to Order Clostridiales, respectively. Three OTUs belonging to Order Clostridiales, Family Erysipelotrichaceae and Family Ruminococcaceae were negatively correlated with piglets ADG (Table 4).

**Table 4:** Discriminant OTUs associate with piglets’ ADG in High dose yeast supplemented group.

Pairwise association with ADG	OTUs
0.63	Genus *Prevotella*
0.62	Genus *Prevotella*
0.58	Order Clostridiales
0.58	Order Clostridiales
0.57	Order Clostridiales
0.56	Family Ruminococcaceae
0.55	Family Ruminococcaceae
0.53	Genus Blautia
0.53	Genus Prevotella
0.53	Order Clostridiales
0.52	Family Ruminococcaceae
0.52	Genus Blautia
0.51	Family Ruminococcaceae
-0.50	Order Clostridiales
-0.50	Family Erysipelotrichaceae
-0.55	Family Ruminococcaceae

## Discussion

Results from this study revealed the impact of *S*. *cerviase* yeast supplementation on growth performance of suckling piglets and on the modulation of their cecum microbial composition. The effect of live yeast supplementation on the growth performance of pigs is controversial. No effect of yeast supplementation on body weight gain was reported in sows [4,33,34], neonatal pigs [35], and weanling pigs [5]. On the other hand, supplementation of pig diets with live yeast was reported to improve feed intake and weight gain in weanling pigs [7,8,9] and to counteract detrimental effect of ETEC or *Salmonella* infection in post-weaning piglets [10,11,12]. Similarly, live yeast supplementation to gestation and lactation sow diets has been shown to improve average daily weight gain of the piglets from yeast fed sows [36,37] possibly due to improved quality and increased level of IgG in colostrum and IgA in the sow milk [4,6], which may have also resulted in increased level of IgG in the serum of piglets due to passive immunity [34]. Our results add to and are in agreement with the later studies demonstrating improved growth rate of suckling piglets orally supplemented with live yeast. This is very important in practice, due to the positive correlation between the weight at weaning and the piglet survival [38]. The mode of action by which live yeast supplementation improves health and performance of pigs in general, and the suckling piglets in particular, is not completely clear. Although yeast components may have mediated a direct immune modulating effect ultimately contributing to improved health and performance [39,40,41]. Yeast supplementation may also have induced changes in the gut microbial population which in turn mediated performance responses [42]. Indeed, the gut microbiota has been associated with modulation of host metabolism [43] and the host-microbiota interaction has been considered a frontier area for livestock science [44].

In our study, Low yeast supplementation had the greatest influence on the cecal microbiota profile of suckling piglets. The Low dose group had a reduced microbial richness compared with the Control group, indicating that live yeast supplementation can modulate the hind gut microbiota of suckling piglets by reducing its variability. Supporting our line of argument, previous studies conducted in suckling and weanling pigs have reported decreased total bacterial count and a reduced diversity of faecal microbial population in young pigs supplemented with live yeast as compared to control piglets [8,45]. In general, an increase of microbial diversity has been associated with a greater microbial ecosystem stability and with an improved health status of the host [46,47]. However, some studies disproved this correlation between biodiversity and ecosystem stability by introducing the concept that microbial ecosystem stability can vary according to host-associated microbiomes upon different environment and stress response, supporting the concept that higher microbial variability does not always represent a favorable condition for the host [48,49]. During the first couple of weeks after birth, piglets have shown an unstable gut microbiota; the alpha diversity indices, which reflect the microbial variability, have been reported to increase markedly until weaning [46], and a stable adult-like microbiota is reached between 10 and 21 days after weaning [46,50,51,52]. Following the concept proposed by Glasl et al. [48] that the microbial stability can vary according to the host condition, we proposed here that live yeast supplementation to piglets during this neonatal period may help to stabilize the gut microbiota and establish a favorable microbial condition in young piglets. This is supported by the better growth performance of yeast treated groups compared to the Control group observed in this study. In fact, in the probiotic strategy it is expected that the probiotic will colonize the intestinal site of the host and, to reduce the pathogens’ development, resulting in reduced microbial variability, as already proposed by other researchers [53,54]. On the other hand, the highly diverse microbiota in the cecum of Control pigs observed in this study could indicate that the gut microbiota in the Control group was subjected to greater instability reflecting the wide variety of bacteria that the piglets were exposed to during suckling period that may have easily established in the gut of the Control pigs in the absence of the yeast probiotic. However further and more robust studies are needed to confirm our hypothesis.

We reported here that the dominant bacterial populations in the cecum of suckling pigs were those belonging to the Firmicutes and to the Bacteroidetes phyla. This is in agreement with previous studies conducted in faeces [46, 55] and colon [53] of suckling piglets, but, apart from that, our results have also shown that the third major dominant bacteria were those that belong to the phylum Actinobacteria. In a previous study, phyla Fusobacteria and Proteobacteria were reported as the third and fourth dominant phyla in the faecal and colon microbial profile of suckling pigs, while phyla Actinobacteria was reported at a lower level (about 0.6–1.5) [46,53]. However, this difference could be ascribed to the different intestinal tract analyzed among studies, indeed our finding on the abundance of Actinobacteria is in agreement with the results reported by Yu et al. (2018) in the cecum of suckling piglets [56]. In our study, a higher relative abundance in Actinobacteria was observed in the Low yeast group compared to control group as already reported by Kiros et al. (2018) [57] using the same yeast strain and dose and by Brousseau et al. (2015) [58] using the *Saccharomyces cerevisiae* subsp. *boulardii* along the hind-gut (cecum and colon) of post-weaning pigs. This may highlight that yeast supplementation selects the Actinobacteria both in pre and post-weaning phases. In addition, phylum Actinobacbacteria has been indicated as the most stable phylum in piglets’ faeces before and after weaning [46]. Having a higher relative abundance of this phyla during suckling period may favored its resiliency after weaning and it may contribute to a greater microbial stability during the weaning transition.

Furthermore, differences at genus level were observed among groups. The Low dose yeast group had a higher relative abundance of *Blautia*, *Eubacterium* and *Collinsella*, bacterial genera which are commonly present in mammalian intestinal microbiota. Previous findings on these genera were mainly related to human and mice and increased abundance of mentioned genera have been linked with good intestinal health of the host [57–63]. For example, *Collinsella* genus belongs to Actinobacteria phylum and has been previously reported to increase in colon and cecum of yeast-supplemented weaned piglets [57] and has been indicated as one of the major utilizer of lactose in human gut and can modulate the virulence and pathogenicity of potential enteric pathogens [59]. *Blautia* genera can ferment different type of carbohydrates to produce acetic acid, lactic acid and ethanol [60] and have been associated with an improvement in glucose metabolism in mice [61]. *Eubacterium* have been reported to produce butyric acid, which is particularly important for colonic homeostasis [62,63]. The divergent response of microbial composition to the yeast dosage has been previously reported [64,65], thus our observation emphasizes again the importance of probiotic dosage in influencing the microbial profile. Indeed, repeated large introductions of a probiotic strain does not always result in an increase of the probiotic effect in modulating the microbial profile [66]. In our study, it was possible to discriminate specific genera which were influenced by the highest yeast dose. Similar to the Low yeast supplementation, the High dose yeast supplementation increased the relative abundance of genera associated with SCFAs production, including *Eubacterium*, *Anaerostipes* which are butyrate producers [63], *Parabacteroides* which are succinate producers and *Phascolarctobacterium* which produce propionate via succinate fermentation [67]. Therefore, it seems that both Low and High dose yeast supplementation improved the level of short chain fatty acid producing bacteria, which may help to promote the piglets’ intestinal health. However, our study did not measure the level of SCFAs in the cecum of the piglets and hence further studies are needed to verify this hypothesis and to associate oral yeast supplementation with modification of the microbial metabolism in piglets’ cecum.

Furthermore, since both the Low and High dose supplemented yeast group showed significantly higher ADG values compared to Control, and as ADG can be influenced by intestinal microbial metabolism, we tested the association between cecum microbial profile and the piglets’ performance. In the Low dose yeast, no positive correlation between cecum microbiota and piglets ADG has been observed, while in the High dose yeast group we observed that *Prevotella* was positively correlated with piglets’ ADG, in agreement with previous findings [68,69]. This result suggests that yeast supplementation may affect the piglets’ ADG through different mechanisms depending on the yeast dose and further elucidation would be needed to confirm this.

The positive correlation between *Prevotella* abundancy and the piglets’ growth performance has been attributed to its ability to process complex dietary saccharides of the diet and favoring monosaccharide uptake by the host [69]. In our study, piglets had no access to creep feed, however, bacteria from genus *Prevotella* can also use monosaccharide such as glucose which may results from the digestion of the milk-derived lactose, to produce SCFAs [70]. The production of SCFAs is one of the mechanisms by which intestinal microbiota can influence and promote the host metabolism and physiology [71] and may explain the positive correlation between *Prevotella* and growth performance observed in our study. Overall, piglets’ performance can be influenced also by sows’ conditions [72], piglet birth weight and litter size [73, 74], piglets feed intake [73], colostrum and milk composition [74, 75], factors which we were not able to control in the present study. Thus, further elucidation of the positive correlation between *Prevotella* and ADG of suckling piglets observed in our study is desirable.

## Conclusion

In conclusion, our results showed that yeast supplementation during sucking period improved piglet performance and shaped the piglet cecum microbiota composition in a dose dipendent way. The lower yeast tested dose was more efficient in modifying the cecal microbial profile reducing the bacterial richness in cecum. Furthermore, both lower and higher doses of yeast promoted the development of bacteria known as SCFAs producers, which may have positively influenced the piglet metabolism resulting in improved performance; however, measuring the level of SCFAs produced is needed to confirm this hypothesis. In addition to this, studies aimed at further elucidation of the mechanisms by which different dose of live yeast supplementation interacts with the host to improve piglet health and growth are desirable in order to promote yeast supplementation as an alternative strategy to increase piglets’ robustness before weaning with an ultimate objective of reducing the use of antibiotic in the swine industry.

## Supporting information

S1 Table Piglets’ weight at the different time points and piglets’ ADG. Piglets were treated by oral gavage with either 5 x10^9^ cfu (Low) dose or 2.5 x 10^10^ cfu (High) dose of live yeast or sterile water (Control) every other day starting from day one of age until weaning. Body weight was recorded at regular intervals and presented either as piglet weight in kilograms or daily weight gain in kilograms.
(XLSX)

S2 Table PLS results for discriminant OTUs associate with piglets’ ADG in Low dose yeast supplemented group. (XLSX)

## Abbreviations

ADG: Average daily gain
ETEC: Enterotoxigenic *Escherichia coli*
NMDS: Non-Metric Multidimensional Scaling Plots
OTUs: Operational Taxonomic Units
PLS: Partial Least Squares
*S. cerevisiae*: *Saccharomyces cerevisiae*
SCFA: Short chain fatty acids
sPLS-DA: Sparse Partial Least Squares Discriminant Analysis
